# Effects of object working memory load on visual search in basketball players: an eye movement study

**DOI:** 10.1186/s40359-023-01488-6

**Published:** 2023-12-19

**Authors:** Qing Nian, Wenping Lu, Ying Xu

**Affiliations:** 1 School of Physical Education, Northeast Normal University, Changchun, 130024 China; 2 Physical Education Department, Jilin University of Finance and Economics, Changchun, 130117 China

**Keywords:** Object working memory, Visual search, Basketball players, Eye movement research

## Abstract

Working memory may affect the athletes’ visual search ability. Objective: This study aimed to examine the differences in the performance of visual search tasks among basketball players of varying sport levels, considering the influence of different object working memory loads. Method: This study recruited forty-two participants who were divided into three groups based on the classification of elite athletes: competitive elite, semi-elite, and novice. Results: Objective working memory load significantly impacts the accuracy of visual search, reaction time, and gaze fixation in basketball players. In the visual search task of the basketball sports scene, the inclusion of object working memory load led to a significant decrease in the accuracy of visual search, a significant increase in reaction time, a significant increase in the number of fixation points, and a more complex gaze trajectory. In a visual search task with object working memory load, the difference in reaction time between basketball players of different sport levels was observed during the search initiation time and scanning time, with higher sport levels associated with shorter reaction times. The effect of object working memory load on the eye movement phase of visual search varied among basketball players of different sport levels. For the novice group, the effect was on the reaction time during the verification phase, while for the semi-elite and competitive elite groups, the effect was on the reaction time during the scanning phase. Conclusion: The effect of object working memory load on visual search varied among basketball players of different sport levels.

## Background

Visual search is the process through which individuals actively seek targets within a complex environment and has emerged as a crucial experimental paradigm employed by cognitive psychologists to investigate selective attention [1–3]. In sports, visual search reflects how athletes process information [4]. Therefore, it is crucial to examine the characteristics of athletes’ visual search during competition and the factors contributing to its development to enhance sports performance [5, 6]. Previous research has shown that athletes of varying sport levels exhibit minimal differences in basic visual abilities, but notable disparities arise in their professional visual abilities. This is evident in the superior visual search ability of elite athletes under professional conditions compared to novices, as evidenced by their adept utilization of visual cues and appropriate visual search strategies [7, 8]. Several studies have consistently shown that elite athletes outperform novices in various aspects, including shorter reaction times, higher efficiency, cleaner eye trajectories, fewer but more focused gaze fixation points, and superior allocation of attentional resources during visual search tasks [9, 10].

Basketball is a sport that necessitates a heightened level of visual search ability. Throughout the game, basketball players must consistently monitor court dynamics, filter out irrelevant information, and precisely identify crucial offensive and defensive focal points. They constantly encounter the challenge of transitioning between offense and defense during gameplay. During offensive maneuvers, players must consistently monitor the positions of their teammates and the ball, while during defensive actions, they must remain attentive to the target of their defense and the ball’s location. In both offensive and defensive scenarios, basketball players must instantaneously observe multiple targets and react swiftly [11]. A study has revealed that experienced basketball players employ a simpler and more effective visual search strategy in comparison to novices [12]. Recent research has indicated that working memory significantly influences visual search. Several studies have demonstrated that maintaining a visual working memory load during visual search results in reduced search efficiency [13, 14]. Working memory refers to a memory system that has a limited capacity for temporarily storing and processing information during cognitive tasks. Specifically, visuospatial templates are also known as visual working memory, which can further be categorized into object working memory and spatial working memory [15, 16]. According to current research, the working memory system retains graphical physical information, such as color and shape, in object working memory, while orientation information is stored in spatial working memory [17–19]. Numerous studies have employed a dual-task paradigm, combining a working memory task with a visual search task, to investigate the impact of working memory on visual search. These studies consistently demonstrate the substantial influence of object working memory on visual search. Research conducted by Castelhano demonstrated that scene context can enhance object search in natural scenes [20, 21]. W Wolfe illustrates that the characteristics of the target object can impact visual search in real scenes [21]. Specifically, the presence of a load on object working memory significantly impairs performance in visual search tasks, resulting in longer search reaction times and decreased accuracy. However, studies have demonstrated that the presence of a load on object working memory does not diminish the efficiency of visual search, irrespective of whether the task employs traditional meaningless images or real scenes [13, 14, 22–26]. In order to gain a more precise understanding of the impact of object working memory on visual search, Malcolm & Henderson categorized visual search into three distinct phases: search initiation time, scanning time, and verification time. Search initiation time, also referred to as the latency of the first eye-hop, denotes the duration from the presentation of the search interface until the completion of the initial gaze fixation; scanning time represents the duration between the onset of the first eye movement and the first fixation on the search target; verification time signifies the duration from the initiation of the first fixation on the search target until the keystroke response, reflecting the time taken by the subject to match the target with the search target. The study conducted by Malcolm & Henderson concluded that segmenting visual search into three stages enables a comprehensive analysis of its features and facilitates a more in-depth examination of visual search based on these features. Malcolm’s study has shown that the specificity of the target cue influences both the activation map, which depicts potential target locations, and the process involved in matching a fixated object to an internal representation of the target. Their research further demonstrated that the combination of target template and contextual constraints has an additive effect in enhancing search efficiency [27, 28].

This study employed a dual-task paradigm that integrated object working memory and visual search. Additionally, eye movement techniques were utilized to categorize the eye movement process of visual search into three distinct phases: search initiation time, scanning time, and verification time, based on the classification method proposed by Malcolm & Henderson. The aim of this study is to identify distinct variations in the visual search processes among basketball players of varying skill levels, while considering different object working memory load conditions. This will be accomplished by examining the eye movement processes associated with visual search. The primary objectives of this investigation are to explore the potential impact of object working memory load on the eye movement processes involved in the visual search task among basketball players of diverse skill levels. Additionally, it aims to analyze the cognitive disparities in visual search between basketball players of varying skill levels, with the intention of differentiating elite athletes from novices. The findings from this study may have implications for the selection of basketball players. The study hypothesized that the effect of object working memory load on visual search was different for basketball players of three different sport levels, competitive elite, semi-elite, and novice, and that there was an expert advantage. Specifically, competitive elite athletes possess a greater visual search advantage compared to both semi-elite athletes and novices.

## Method

### Subjects

A total of forty-two subjects were recruited for this study and subsequently classified into three distinct groups based on the criteria for elite athletes [29]: the competitive elite group, the semi-elite group, and the novice group. The classification criteria for the competitive elite athletes group required more than 8 years of professional training. The semi-elite athlete group encompassed individuals with 4–8 years of professional training who met the corresponding classification criteria. The novice group consisted of individuals with no prior professional training experience. According to China’s athlete classification system, competitive elite athletes were categorized as national level athletes or above, while semi-elite athletes were classified as national level 2 athletes or above. Novices, on the other hand, do not possess any athletic level. The competitive elite athletes were recruited from the Jilin Provincial Basketball Team, which comprises athletes at the national level. The semi-elite athletes and novices were recruited from Northeast Normal University (China). The semi-elite athletes belong to the Northeast Normal University basketball team and possess a national level 2 athletic rating. The novices were enrolled in the basketball specialized class and do not possess any athletic level. The subjects were based on a priori Power analysis using G*Power 3.1.9.7with effect size of 0.35, alpha of 0.05, power of 0.85. After calculation, 33 subjects were sampled to meet the analysis requirements. After screening, a total of 42 subjects were enrolled in the test. All participants were male, had normal or corrected vision, and were right-handed. Participants who met any of the following criteria were excluded: (1) had experienced a major illness (e.g., a fracture) within the last year; (2) had a physical illness (e.g., heart disease); (3) had a mental illness (e.g., anxiety); (4) had an eye disease such as color blindness or color weakness, etc.; (5) had participated in similar experiments before. The Ethics Committee of Northeast Normal University approved this experiment. All study subjects signed informed consent and agreed to the experimental (basic information of the subjects was shown in Table 1).

**Table 1:** Subjects characteristics (M ± SD)

Group	Number	Age	Working memory capacity
Competitive elite group	11	24.46 ± 1.43	4.75 ± 0.93
Semi-elite group	15	22.33 ± 1.05	5.07 ± 0.26
Novice group	16	21.38 ± 1.67	5.18 ± 0.75

Note: M: mean; SD: standard deviations

### Experimental design

This experiment employed a dual-task experimental design where each participant performed a visual search task under varying object working memory load conditions. The experimental design consisted of a 3 (sport level: competitive elite group, semi-elite group, novice group) × 3 (object working memory load: 0, 2, 4) matrix. The sport level was considered as a between-group factor. The dependent variables comprised: (1) the percentage of participants correctly completing the visual search task, which reflects their ability to find targets within a limited time frame; (2) the total reaction time of visual search, indicating the participants’ overall reaction ability, where a faster reaction time suggests quicker target detection; (3) the reaction time of the search initiation time, scanning time, and verification time, representing the reaction times for each specific phase to identify differences in athletes’ reaction times across phases; (4) the number of gaze fixation points and eye movement trajectory during visual search, reflecting search efficiency, with fewer gaze fixation points and simpler eye trajectories indicating higher efficiency.

### Experimental materials

The visual search task was derived from images of National Basketball Association, FIBA Basketball World Cup and other games on the Internet. The images were manipulated using Photoshop CS3, which involved simple resizing to 1024 × 768 pixels. The visual complexity of the images was then measured using the calculation method proposed by Rosenholtz [30]. This method primarily measured feature congestion (FC) and subband entropy (SE) as indicators. Images with intermediate FC and SE values (FC: 3.24 ± 0.32; SE: 3.32 ± 0.15; moderate positive correlation between FC and SE: r = 0.48, *p* < 0.001) were selected, resulting in a total of 158 images chosen as materials for the visual search task. 79 images were randomly selected, and the object within each image was designated as the correct search target (representing the unique component of the picture). Additionally, some of the remaining basketball images that were not chosen as experimental material were used as incorrect search targets (ensuring they did not appear in the initial pool of 158 images). All search target images were resized to 50 × 50 pixels.

The experimental material for the object working memory load consisted of geometric figures constructed using seven easily distinguishable colors. The seven easily distinguishable colors included red (RGB: 255, 0, 0), orange (RGB: 255, 165, 0), yellow (RGB: 255, 255, 0), green (RGB: 0, 255, 0), blue (RGB: 0, 0, 255), black (RGB: 0, 0, 0), white (RGB: 255, 255, 255). These colors were randomly combined with seven geometric shapes (circle, triangle, square, prism, hexagon, cross, cylinder) to create 49 geometric figures, all measuring 50 pixels x 50 pixels. For both object working memory loads of 2 and 4, four geometric figures with distinct shapes were simultaneously presented. However, only two colors were available and evenly distributed among the four figures for the object working memory load of 2, while four colors were used for the object working memory load of 4. The four geometric figures were individually presented at predetermined locations on the screen. The coordinates of these presentation locations are provided in Table 2.

**Table 2:** Geometric figures position coordinates

Number of geometric figures	Coordinates
Four	(376, 248) (648, 248) (376, 520) (648, 520)

Note: Coordinate system with the bottom left corner of the screen as the origin, horizontal to the right direction for the x-axis, the range of values was 0-1024pixel; vertical upward direction for the y-axis, the range of values was 0-768pixel

### Experimental instrumentation

The experimental materials and stimuli were displayed on a 17-inch Liquid Crystal Display (LCD) (E1715S, DELL, USA) with a gray screen color (RGB: 147, 147, 147). The LCD had a primary screen resolution of 1280 × 1024 pixels and a refresh rate of 60 Hz. Eye movement data was acquired using an eye-tracker (Eyelink 1000plus) with a sampling frequency of 1000 Hz and a visual distance of 75 cm. The stimulus material was presented to the subject on the LCD monitor, while another computer was used to record the subject’s experimental eye movement data. The subject’s eye position was required to be aligned with the center of the LCD. The experiments were programmed and controlled using E-prime 3.0 software. The entire experiment was conducted in a laboratory with moderate lighting conditions and no external environmental interference.

### Experimental procedure

**Fig. 1:**
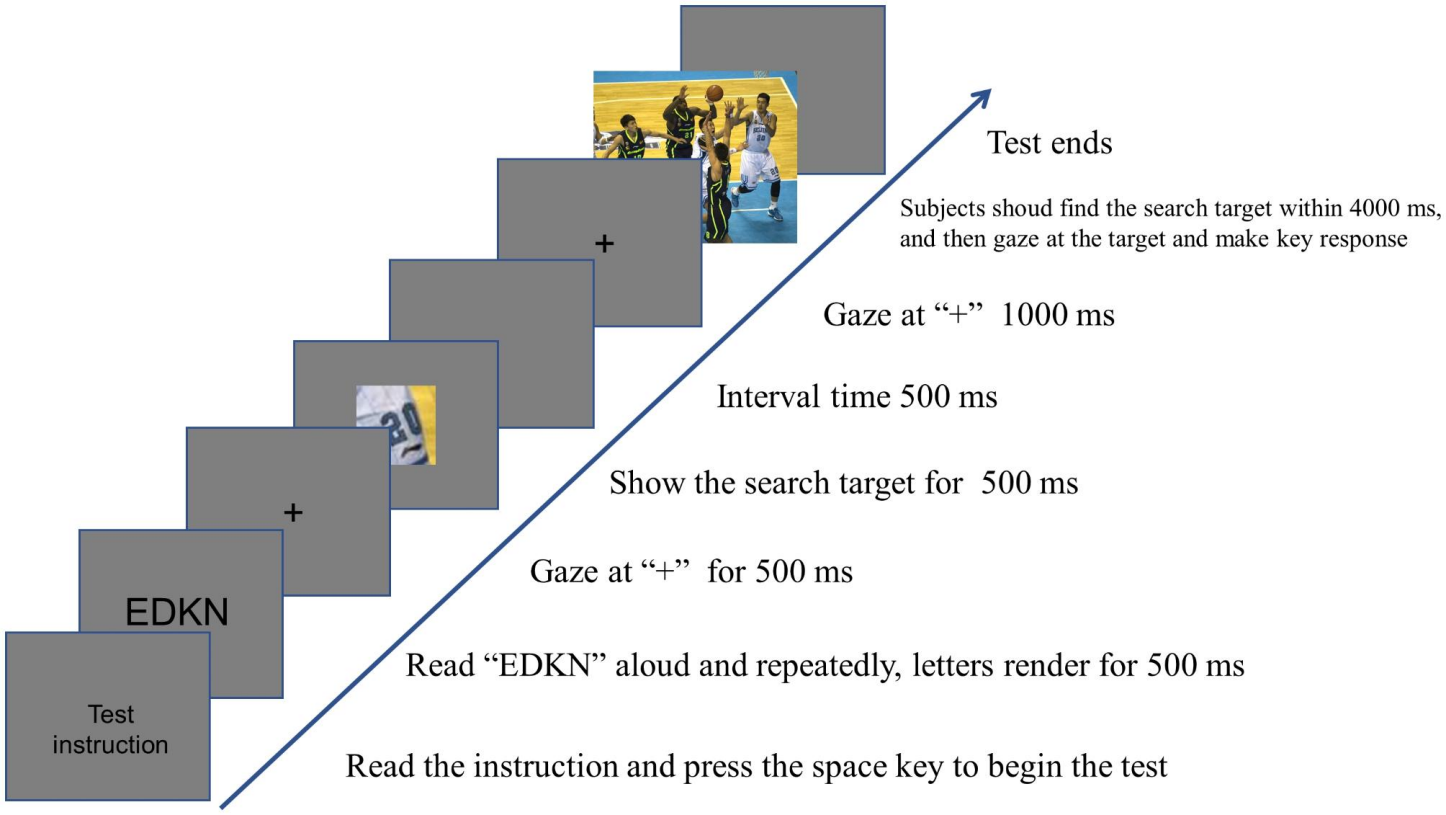
Flow chart of visual search experiment without object working memory load

**Visual search experiments were conducted without object working memory load.** These experiments consisted of a single task, as displayed in Fig. 1. To prevent any interference with the encoding of verbal working memory during the experiment, four random uppercase letters are displayed on the screen before the start of each trial [31]. The experimental instructions were presented initially, followed by the presentation of four randomized capital letters for 500 ms. Subjects were instructed to read the letters aloud twice before the formal experiment commenced. Following the initiation of the formal experiment, a “+” point appeared on the center of the screen for 500 ms. Subsequently, the search target was displayed for 500 ms, followed by a 500 ms blank screen. Afterward, the “+” point reappeared in the center of the screen for 1000 ms, prompting the subject to focus on the image. The image of the scene to be searched was then presented for 4000 ms, during which the subject had to locate the search target and respond by pressing the “D” key within the designated time frame. If the target did not appear, the subject was instructed to press the “K” key. Failure to press any key within the specified time was considered an error. After the key response, the screen became blank, and the subject proceeded to the next experiment by pressing the “space” key. In the object working memory loading condition, subjects initially completed four pre-experiments to familiarize themselves with the task. After demonstrating proficiency, they proceeded to the formal experiments, which consisted of a total of fifty trials.

**Fig. 2:**
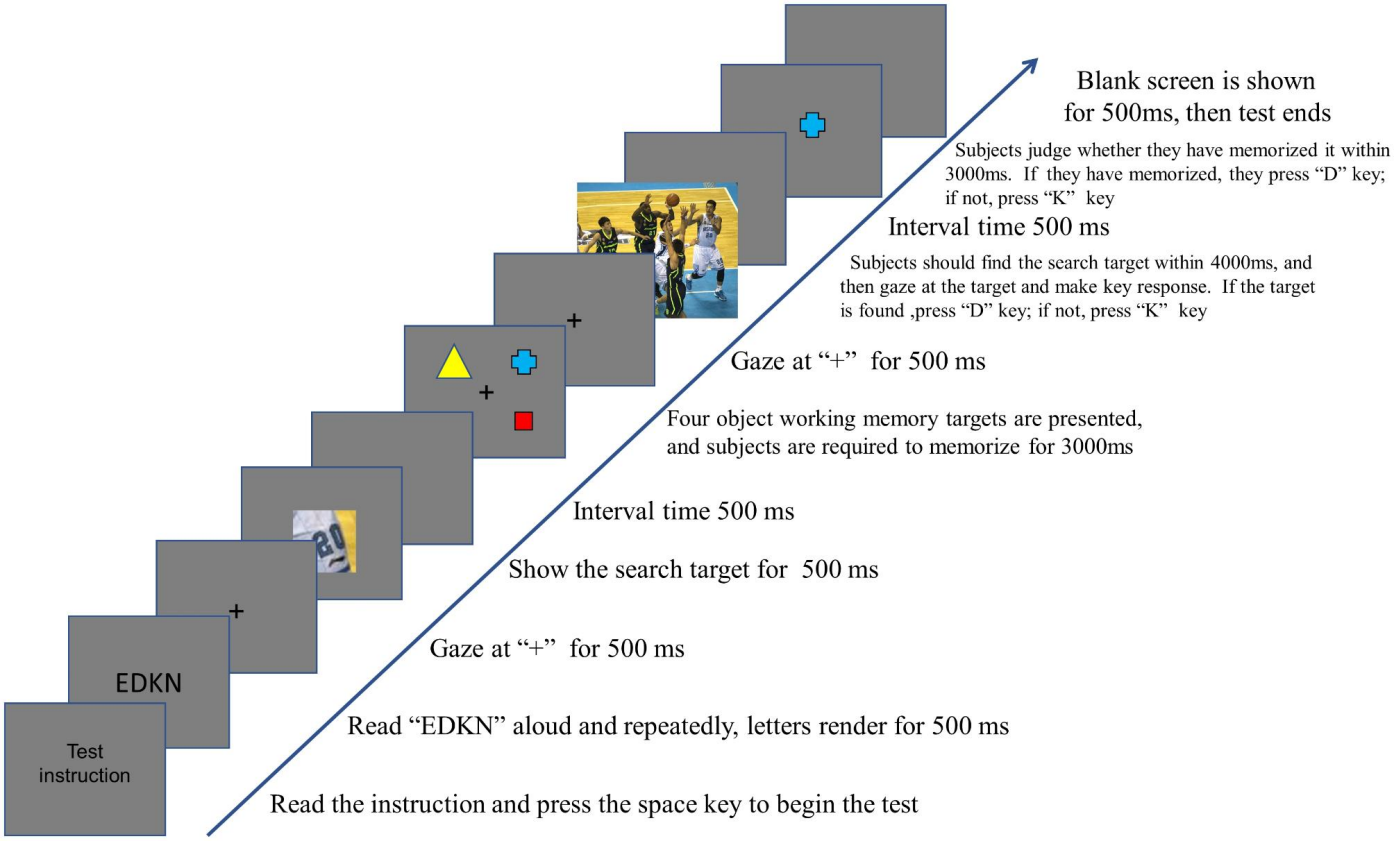
Flow chart of visual search experiment with object working memory load of 4

**Visual search experiments were conducted with an object working memory load of 2 or 4.** Both the visual search experiments with an object working memory load of 2 or 4 were dual-task experiments, combining an object working memory task with a visual search task. Figure 2 illustrates the sequential flow of the visual search experiment with an object working memory load of 4. Following the instructions, four randomized capital letters were presented for 500 ms. The subjects were instructed to read them aloud clearly twice before commencing the experiment. The experiment began with the presentation of a “+” symbol at the center of the screen for 500ms. Next, the search target was displayed for 500ms, followed by a 500ms interval. Subsequently, an empty screen with four random geometric figures in specified positions was shown for 3000ms. Afterward, the “+” symbol reappeared at the center of the screen for 1000ms. Following this, a picture of the scene to be searched was presented for 4000ms. During this time, the subject had to locate the search target and press the “D” key. If the target did not appear, the subject was instructed to press the “K” key. Failure to press the key within the specified time was considered an error. Following the key press, an empty screen with a 500ms interval displayed a geometric figure for 3000ms. The subject was then required to determine whether the geometric figure had just appeared within that time period. If the target appeared, the subject pressed the “D” key. If no target appeared, the subject pressed the “K” key. Failure to press the key within the specified time was considered an error. In the object working memory loading condition, subjects initially completed four pre-experiments to familiarize themselves with the task. After demonstrating proficiency, they proceeded to the formal experiments, which consisted of a total of fifty trials.

All subjects completed three visual search experiments sequentially: one without object working memory load, one with an object working memory load of 2, and one with an object working memory load of 4. A two-minute break was provided between each experiment. Prior to each experiment, the subjects were briefed on the experimental process and the necessary precautions. Subsequently, the subject’s head was secured using a U-shaped forehead rest. The laboratory staff assisted in connecting the identification eye movement recorder and conducting a nine-point calibration of the subject’s eyes to ensure compliance with the standard before commencing the experiment.

### Statistical analysis

The eye movement data were analyzed using the Data Viewer analysis program to identify the three stages of the subjects’ visual search process. Subsequently, the data were imported into SPSS 22.0 for statistical analysis. Specifically, MNAOVA was employed to analyze the collected dependent variable data. All means and standard deviations (SD) were statistically analyzed using standardized statistical methods. The Shapiro–Wilk test was used for the normal distribution of the data, and Levene’s test was used for the homomorphic distribution. Partial Eta squared (η²) was used to calculate effect sizes for significant main effects and interactions.

## Results

All subjects were recruited and underwent a digital span test to primarily assess any differences in their working memory capacity. However, no significant difference in working memory capacity was found among the three groups of subjects (F (2,39) = 1.401, *p* = 0.258). The accuracy rate of visual search, total reaction time, search initiation time, scanning time, verification time, and number of gaze fixation points were recorded for each of the three groups. A comprehensive analysis of these metrics is provided in the subsequent sections.

### The correct rate of visual search

**Fig. 3:**
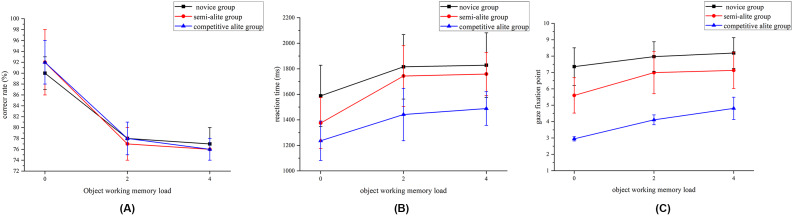
Schematic diagrams of the changes in the main indicators of visual search (**A**: correct rate; **B**: total reaction time; **C**: gaze fixation point)

The correct rate of visual search represents the proportion of successful experiments where subjects accurately located the target object in the scene image and provided the correct keystroke response. MANOVA was conducted to analyze the correct rate of the visual search task. The main effect of sport level was not significant (F (2,39) = 0.098, *p* = 0.907, η²=0.005). However, there were significant main effects of object working memory load (F (2,78) = 309.09, *p* < 0.001, η² = 0.888). The visual search correct rate without object working memory load (91.49 ± 5.35) was significantly higher than the visual search correct rate for object working memory load of 2 (77.61 ± 3.55) (*p* < 0.001) and object working memory load of 4 (76.27 ± 3.02) (*p* < 0.001). However, there was no significant difference between the visual search correct rate for object working memory load of 2 (77.61 ± 3.55) and object working memory load of 4 (76.27 ± 3.02) (*p* = 0.079). There was no significant interaction between sport level and object working memory load (F (4, 78) = 0.800, *p* = 0.529, η² = 0.211). The results were shown in Fig. 3A.

### Total reaction time of visual search

The total reaction time refers to the duration between the presentation of the search scene image and the moment the subject successfully locates the target and provides a correct keystroke response. MANOVA was employed to analyze the total reaction time for visual search, revealing a significant main effect of sport level (F (2, 39) = 17.830, *p* < 0.001, η² = 0.478). The competitive elite group exhibited significantly shorter reaction times (1388.82 ± 165.27) compared to the novice (1744.18 ± 213.58) (*p* < 0.001) and semi-elite groups (1625.75 ± 197.31) (*p* < 0.001), with no significant difference between the novice and semi-elite groups (*p* = 0.113). The main effect of object working memory load was significant (F (2, 78) = 31.575, *p* < 0.001, η² = 0.447). The total reaction time for visual search without object working memory load (1399.84 ± 196.59) was significantly shorter than that for visual search with object working memory load (1679.45 ± 209.38) (*p* < 0.001). However, there was no significant difference in total reaction time for visual search with object working memory load of 2 (1666.87 ± 235.21) or load of 4 (1692.04 ± 247.13) (*p* = 0.241). There was no significant interaction between sport level and object working memory load (F (4, 78) = 1.016, *p* = 0.392, η² = 0.117). The results were shown in Fig. 3B.

### The number of gaze fixation points of visual search

MANOVA was used to analyze the number of gaze fixation points for visual search, revealing a significant main effect of sport level (F (2, 39) = 112.850, *p* < 0.001, η² = 0.853), as well as a significant main effect of object working memory load (F (2, 78) = 27.051, *p* < 0.001, η² = 0.410). There was a significant interaction between sport level and object working memory load (F (4, 78) = 2.97, *p* = 0.03, η² = 0.116). A simple effects analysis was conducted, revealing that the difference in the number of visual search gaze fixation points across object working memory load conditions was not significant for the novice group (*p* = 0.217). In the semi-elite group, the difference in the number of gaze fixation points for visual search across different object working memory load conditions was significant (*p* < 0.01). There were significantly fewer gaze fixation points for visual search without object working memory load (5.60 ± 1.08) compared to the object working memory load of 2 (6.99 ± 1.29) (*p* < 0.001) and object working memory load of 4 (7.13 ± 1.11) (*p* < 0.001). However, there was no significant difference in the number of gaze fixation points for visual search between object working memory load of 2 (6.99 ± 1.29) and load of 4 (7.13 ± 1.11) (*p* = 0.241). In the competitive elite group, there was a significant difference in the number of visual search gaze fixation points across different object working memory load conditions (*p* < 0.001). The gaze fixation points without object working memory load (2.95 ± 0.13) were significantly lower than the gaze fixation points for object working memory load of 2 (4.11 ± 0.30) and object working memory load of 4 (4.81 ± 0.68) (*p* < 0.001). Significant differences in the number of gaze fixation points were observed among basketball players of different sport levels in all experimental conditions, with the novice group having the highest number of gaze fixation points, followed by the semi-elite group, and the competitive elite group. The results were shown in Fig. 3C.

### The process of visual search

**Fig. 4:**
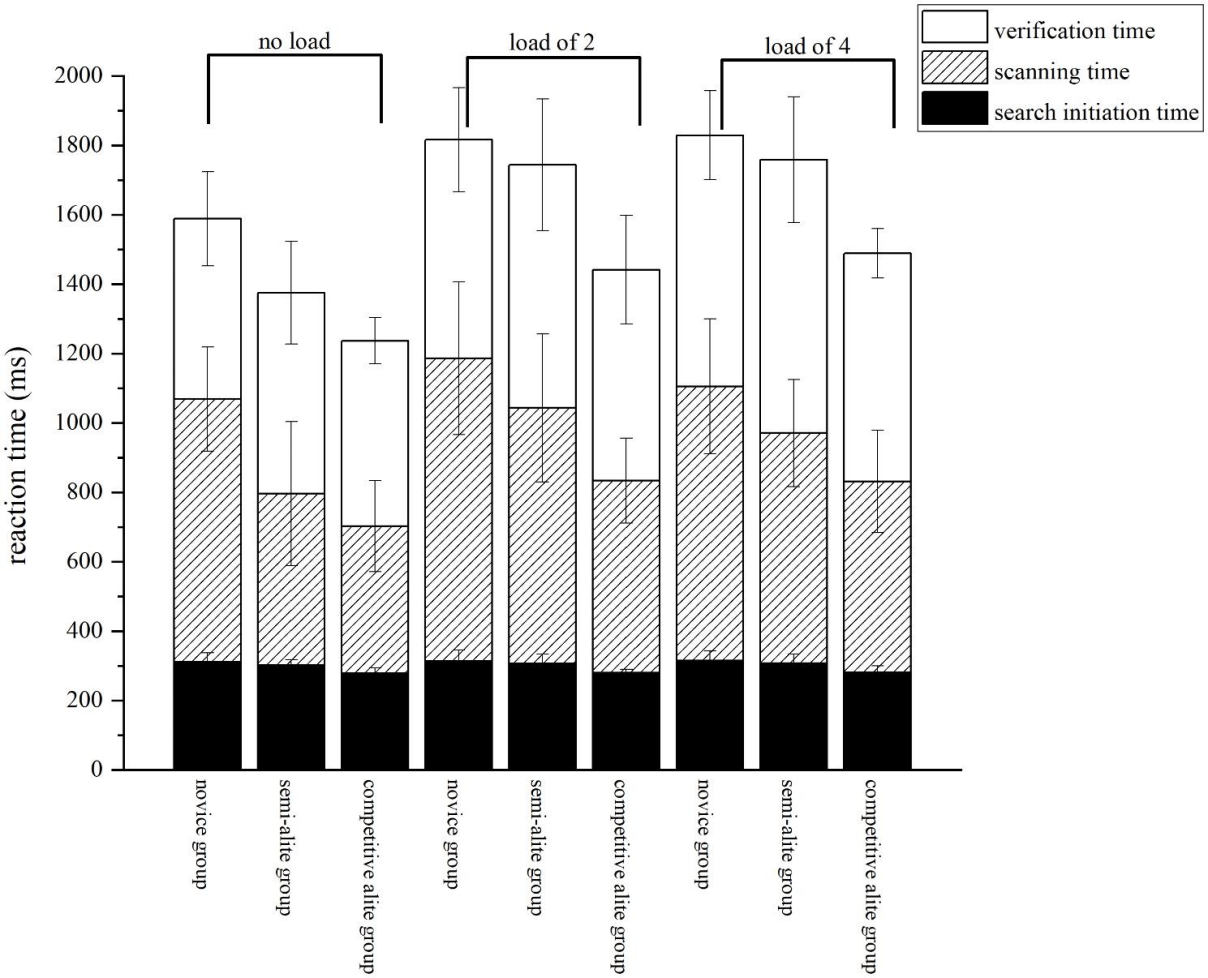
Duration of each phase during visual search response under different object working memory load conditions

The eye movement technique was employed to divide the entire visual search process into three stages: search initiation time, scanning time, and verification time. Search initiation time, also referred to as the first eye-hop latency, represents the duration from the presentation of the search interface to the completion of the first gaze fixation point. Scanning time denotes the duration between the first eye jump and the initial gaze at the search target. Verification time signifies the duration from the first gaze at the search target to the keystroke response, indicating the time taken by the participants to match the target with the search target. The durations of these stages in the visual search process under varying object working memory load conditions are illustrated in Fig. 4. A more comprehensive analysis of each stage of the visual search will be provided in subsequent sections.

**Search initiation time.** The search initiation time reflects the ease with which subjects comprehend the scene from the search screen and make a decision regarding eye movement. Revealing a significant main effect of sport level, F (2, 39) = 11.103, *p* < 0.001, η² = 0.363. The competitive elite group (279.95 ± 18.31) exhibited a significantly faster reaction time in the search initiation phase compared to both the novice group (312.75 ± 29.58) (*p* < 0.001) and the semi-elite group (304.64 ± 29.12) (*p* < 0.001). However, there was no significant difference in the reaction time during the search initiation phase between the semi-elite group (304.64 ± 29.12) and the novice group (312.75 ± 29.58) (*p* = 0.621). The main effect of object working memory load did not reach significance, F (2, 78) = 0.451, *p* = 0.642, η² = 0.010. Similarly, the interaction between sport level and object working memory load was not significant, F (4, 78) = 0.013, *p* = 0.753, η² = 0.001.

**Scanning time.** The scanning time represents the process by which subjects search for targets. A significant main effect of sport level was observed, F (2, 39) = 20.701, *p* < 0.001, η² = 0.515. As sport level increased, the reaction time for the scanning process decreased, and the difference between the two groups was statistically significant (*p* < 0.05). Likewise, a significant main effect of object working memory load was found, F (2, 78) = 10.782, *p* < 0.001, η² = 0.217. The reaction time for the scanning process without object working memory load (637.03 ± 197.84) was significantly faster than the reaction time for object working memory load of 2 (721.04 ± 211.39) (*p* < 0.01) and object working memory load of 4 (667.92 ± 187.35) (*p* = 0.02). However, the interaction between sport level and object working memory load did not reach significance, F (4, 78) = 1.090, *p* = 0.371, η² = 0.021.

**Verification time.** The verification time indicates the duration it took for the subject to confirm whether the gaze object was the search target or not. There was no significant main effect of sport level, F (2, 39) = 2.521, *p* = 0.094, η² = 0.114. However, a significant main effect of object working memory load was found, F (2, 78) = 22.712, *p* < 0.001, η² = 0.368. The reaction time for the verification time without object working memory load (544.40 ± 123.13) was significantly faster than the reaction time for object working memory load of 2 (646.04 ± 179.89) (*p* < 0.01) and object working memory load of 4 (723.37 ± 157.89) (*p* = 0.01). Additionally, the interaction between sport level and object working memory load did not reach significance, F (4, 78) = 0.481, *p* = 0.753, η² = 0.011.

## Discussion

**Fig. 5:**
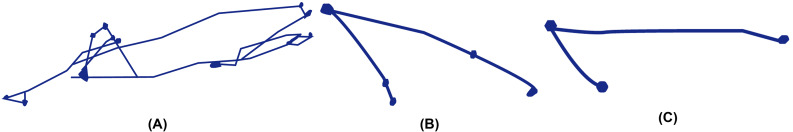
Eye movement trajectories of basketball players with different sports levels searching for the same image (**A**: novice group; **B**: semi-elite group; **C**: competitive elite group)

In this study, all participants engaged in a visual search task with object working memory loads of 0, 2, and 4. Through the analysis of the correct rate, reaction time, and number of gaze fixation points in the visual search task, it was observed that the inclusion of object working memory load led to a decrease in the correct rate of the visual search task, an increase in reaction time, an increase in the number of gaze fixation points, and a more complex eye movement trajectory. These findings support our hypothesis that competitive elite athletes exhibit a visual search advantage compared to semi-elite athletes and novices. Several factors may account for this outcome. Firstly, resource allocation played a role. The visual search experiment without object working memory load was a single-task experiment, whereas the experiment with the addition of object working memory load became a dual-task experiment. In the dual-task experiment, subjects had to allocate attention and memory resources to both the search target and the geometric image. However, individuals have limited resources that need to be reallocated when attention is demanded by both tasks. It is important to note that attention has a significant impact on an individual’s memory. Allocating more attention to a stimulus leads to deeper processing, resulting in a more profound memory that is less prone to fading [32, 33]. However, the passage of time can also contribute to memory decay. Thus, when assessing memory decay, it is crucial to consider the impact of time. In this study, visual search experiments were conducted with an additional object working memory load, which influenced the subjects’ final scores. As a result, subjects may have allocated their attentional resources to object working memory, leading to a reduction in attentional resources available for the visual search task. Consequently, the depth of processing of the search target decreased. This allocation of attentional resources could cause subjects to repeatedly compare what they see with what they remember during the visual search process or engage in repetitive target searching within the images. These factors contribute to lower correct rates, longer reaction times, a greater number of gaze fixation points, and more complex gaze trajectories. The second factor to consider is the potential occurrence of retroactive interference [34, 35]. Retroactive interference refers to the phenomenon where post-learning content influences pre-learning content [36, 37]. In the visual search experiments involving object working memory load, both visual search targets and geometric figures were presented. Consequently, the recognition of geometric figures interfered with the memory of the search targets. Figure 5 displays the eye movement trajectories of athletes at different sport levels while searching for the same picture under a working memory load of 4. The novice, semi-elite, and competitive elite groups are shown from left to right. The eye movement trajectories of the novice group appear complex and disorganized, whereas those of the competitive elite and semi-elite groups are simple and straightforward. This observation suggests that athletes with higher sport levels possess superior abilities in strategy-based searching and information integration.

Further analysis revealed that object working memory load decreased the accuracy of visual search, but there was no significant difference in the impact of high or low object working memory load on accuracy. In the experimental task, the time required for memory retention was 3000 ms shorter in the 0 load condition compared to the 2 and 4 load conditions. Therefore, the observed decline in behavioral performance in the 2 and 4 memory load conditions, when compared to the 0 load condition, could potentially be attributed to a time delay. The results depicted in Fig. 3 indicate that memory loads 2 and 4 were both lower than load 0, but the difference was not significant. This suggests that a time delay of 3000ms may not have an impact on the decline in behavioral indicators. There was no significant difference in the accuracy of visual search among the three groups of athletes, regardless of the presence or absence of object working memory load. This suggests that the accuracy of visual search in basketball scenarios cannot be utilized as a criterion for selecting basketball players. Similarly, the object working memory load led to a significant increase in response time for the visual search task, but there were no significant differences between the reaction times of visual search with an object working memory load of 2 or 4. The results for gaze fixation points differed from the accuracy and reaction time as object working memory load only significantly increased the number of gaze fixation points in the competing elite and semi-elite groups. However, there was no significant difference in the number of gaze fixation points in the novice group across the three load conditions. The semi-elite group had a higher number of gaze fixation points with both the object working memory load of 2 and 4, but there was no significant difference in the number of gaze fixation points between the object working memory load of 2 and 4. In contrast, the number of gaze fixation points in the competitive elite group exhibited a gradual increase with an increasing working memory load of the object. Furthermore, we observed significant differences in the number of gaze fixation points among basketball players of different skill levels across all experimental conditions, with the novice group having the highest count, followed by the semi-elite group, and then the competitive elite group. These findings suggest that the number of gaze fixation points in the visual search task of basketball scenes can serve as an indicator for selecting basketball players. One possible explanation for the differences in gaze fixation points among the three groups is that the novice group already exhibited irregular search patterns in terms of gaze fixation points in the specialized condition. Therefore, increasing the object working memory load would not significantly impact the number of gaze fixation points. In contrast, the semi-elite and competitive elite groups enhanced their object working memory in order to improve their ability to memorize the search target. This increase in memory load would consume a portion of their cognitive resources, resulting in a higher number of gaze fixation points. The impact of varying object working memory loads on the accuracy of visual search in basketball players at different skill levels remained consistent. This consistency can be attributed to the inherent difficulty of the visual search task when high object working memory load settings are employed [38]. Through the calculation of the correct rate under different object working memory loads, it was determined that all subjects in both object working memory load experiments achieved a correct rate of approximately 61%, which is only marginally higher than the random probability. Furthermore, interviews conducted with the subjects consistently indicated that the object working memory task was challenging, often requiring them to rely on intuition for making judgments. Future studies should focus on investigating the appropriate type of object working memory load that can effectively differentiate between basketball players of varying skill levels. Further analysis revealed a distinct expert advantage within the competitive elite group, particularly evident in the metrics of gaze fixation point and reaction time. This advantage can be attributed to the extensive on-court experience of the competitive elite group, resulting in their exceptional familiarity with basketball game scenarios and their ability to swiftly and accurately locate the desired target.

This study employed the eye movement index to divide the visual search process into search initiation time, scanning time, and verification time. The aim was to identify distinct differences in the visual search process among basketball players of varying skill levels under different loading conditions. The results indicated significant differences in the search initiation time and scanning time among basketball players of varying skill levels. During the search initiation time, the competitive elite group exhibited significantly faster response times compared to the semi-elite and novice groups. In terms of scanning time, higher exercise levels were associated with shorter response times in the scanning phase, and significant differences were observed between the two groups. The search initiation time serves as an indicator of the subject’s speed in mobilizing resources from the end of the gray screen to the appearance of the first gaze fixation point response. The significant difference in speed of resource mobilization between the competitive elite group and the novice and semi-elite groups implies a strong association between this speed and competitive basketball training. Elite athletes undergo extensive training, resulting in accelerated brain reaction speed and the ability to rapidly mobilize cognitive resources. This enables their brain and body to swiftly transition into a competitive state, ready for intense and fierce competition [39, 40]. However, the novice group did not exhibit a similar reaction speed, highlighting the crucial disparity in resource mobilization speed between the novice and competitive elite groups. The scanning time represents the central stage of visual search, assessing the subjects’ search strategies and abilities. The duration of search time in this stage exhibits a strong correlation with the familiarity of the search scene. The three groups of subjects in this study had varying years of training, with the novice group having the shortest duration, the semi-elite group in the middle, and the competitive elite group having the longest duration. Consequently, their familiarity with the basketball scene is expected to follow an increasing order, aligning with the findings of our study. No significant differences were observed in the verification time among the three groups of subjects. This lack of difference can be attributed to the subjects’ similar working memory capacity, resulting in comparable abilities to remember the target. The study also revealed inconsistent effects of object working memory load on the eye movements during a visual search task among basketball players at different skill levels. Among the novice group, object working memory load solely influenced the verification time during visual search, leading to a noteworthy increase in reaction time for verification. In contrast, the semi-elite and competitive elite groups experienced a notable increase in reaction time for scanning when subjected to object working memory load. However, a significant increase in reaction time for verification only occurred when the object working memory load of 4. Our findings may have practical implications in real basketball games. For instance, an increase in irrelevant stimuli can influence visual search, thereby impacting the performance of basketball players. However, this study has certain limitations. Specifically, we solely focused on examining the impact of object working memory on visual search, neglecting the effect of spatial working memory. Future research should address the effect of spatial working memory on visual search, which will be our next research objective.

## Conclusion

The impact of object working memory load on visual search varied among basketball players at different skill levels. This finding can serve as a basis for selecting basketball players and contribute to the identification of higher-level athletes.

## Data Availability

The datasets generated and analysed during the current study are not publicly available due to privacy reasons, but are available from the corresponding author on reasonable request.
